# Threat-related corticocortical connectivity elicited by rapid auditory looms

**DOI:** 10.1038/s41598-025-30552-x

**Published:** 2025-12-12

**Authors:** Karolina Ignatiadis, Roberto Barumerli, Gustavo Deco, Brigitta Tóth, Robert Baumgartner

**Affiliations:** 1 Acoustics Research Institute, Austrian Academy of Sciences, Dominikanerbastei 16, Vienna, 1010 Austria; 2 Center for Brain and Cognition, Computational Neuroscience Group, Faculty of Medicine and Life Science, Universitat Pompeu Fabra, Roc Boronat 138, Barcelona, Spain; 3 Institució Catalana de la Recerca i Estudis Avançats (ICREA), Passeig Lluís Companys 23, Barcelona, Spain; 4 Centre for Eudaimonia and Human Flourishing, Linacre College, University of Oxford, Oxford, UK; 5 Institute of Cognitive Neuroscience and Psychology, HUN-REN Research Centre for Natural Sciences, Magyar tudósok körútja 2, Budapest, 1117 Hungary

**Keywords:** Auditory looming bias, Distance motion perception, Brain connectivity, Fear, Hazard protection, Dual pathway model, Perception, Network models

## Abstract

While sounds of approaching objects are generally more salient than those of receding ones, the traditional association of this auditory looming bias with threat perception is subject to debate. Differences between looming and receding sounds may also be learned through non-threatening multisensory information or influenced by confounding stimulus characteristics. To investigate, we analyzed corticocortical connectivity patterns from electroencephalography, examining the preferential processing of looming sounds under different attentional states. To simulate rapid distance changes, we used complementary distance cues, previously studied in the looming bias literature. We observed crucial involvement of frontal cortical regions typically associated with threat and fear responses. Our findings suggest an underlying bias within the ventral ’what’ stream rather than within the dorsal ’where’ stream in auditory information processing, even when the participants’ task was solely focused on the discrimination of movement direction. These results support the idea, that the perceptual bias towards looming sounds reflects an auditory threat detection mechanism, while offering insights into the neural function involved in processing ecologically relevant environmental cues.

## Introduction

If a car is approaching from a distance, its timely detection and avoidance can be essential to our survival. It is presumably to improve warning capacity that approaching stimuli are more salient than receding ones. This perceptual asymmetry, often referred to as the auditory looming bias, has been found to be present across species^1–5^ and ages^6–9^, making it a rather universal trait. As an effective warning mechanism, the looming bias should have the capacity to readily capture attention and be rather universal across cue types. Corroborating this hypothesis, signatures of the bias have indeed been found across attentional states and auditory distance cue types; they were present already at the level of Heschl’s gyrus (HG), housing the primary auditory cortex^10^. Yet, the bias’ relationship to threat detection remains a hypothesis, and discrepancies in behavioral performance, timing, and attentional amplification suggest that there are differences in cortical processing depending on these factors.

The notion of stimulus-specificity has frequently been put forth about the selective advantage of the looming bias and its function as a warning mechanism for organisms facing potential collisions with sound sources. It tends to be observed more consistently in response to stimuli with a natural overtone structure, in contrast to Gaussian white noise stimuli, which sound arguably more artificial^4,9,11^. However, studies have also demonstrated looming biases in response to noise stimuli when accounting for the natural acoustic filtering properties of listeners^12,13^. This suggests that the absence of natural spatial cues, rather than the source’s identity, may be responsible for the failure to elicit the bias in certain cases. Additionally, some investigations of looming bias have employed auditory distance changes as short as 10 ms^10,12,13^, prompting questions about the identity and ecological validity required to evoke this effect.

From a neuroimaging perspective, increased amygdala activation in response to slowly rising sound intensities has been an important argument for the warning function of the bias^14^. Apart from HG and amygdala, functional magnetic resonance imaging (fMRI) has highlighted the involvement of the temporal plane, superior temporal sulcus (STS), prefrontal cortex (PFC) and inferior parietal lobule (IPL) in the preferential processing of looming sounds^14,15^. In general, auditory stimuli have been hypothesized to follow two parallel cortical processing streams: one follows a ventral and the other a dorsal path^16^. Originally stemming from visual research, the dorsal pathway is associated with spatial perception (“where”), while the ventral stream is associated with object identification (“what”)^16,17^. The IPL, thought to play a crucial role in spatial hearing^18,19^ and sound motion processing in particular^20^, is part of the dorsal auditory pathway, whereas the STS and PFC belong to the ventral pathway. Based on these findings, the looming bias circuit emerges as an extended distributed cortical network.

Besides the mere activation increase induced by looming sounds, one crucial aspect is the way in which the involved regions interplay. This question can be addressed through functional connectivity investigations^21^; namely computational methods exploring the information exchange among regions of interest (ROIs). Unlike structural connectivity, which describes anatomical connections linking sets of neural elements, functional connectivity is dynamic in nature. It represents changes in statistical interdependencies between or among brain regions, within a specific time interval and connected to an event of interest. The observed brain regions that are found to contribute the most with respect to connectivity, relative to the others or the combinations thereof, are defined as functional hubs. Those are also dynamic and may deviate from an anatomical definition, as they can be a part of different functional clusters^22^. Findings arising from available connectivity analyses of the auditory looming bias circuit are inconclusive: a study based on intensity ramps argues that top-down directional causal influence from PFC to HG enhances the processing of looming versus receding sounds^23^, while prior investigations on spectral stimuli argue for a bottom-up, temporofrontal connectivity^13^.

Functional connectivity methods employed in previous research focus on a bidirectional analysis process, albeit relying on a small preselection of brain regions (Granger^24^, Phase Transfer Entropy – PTE^25^). Although insightful regarding the interplay of the considered ROI pairs, alternative methods may offer an approach that is closer to a network structure. Those are nevertheless limited by either the number of regions that can be considered (conditional Granger Causality^26^), the dimensionality (multivariate Granger^27^) or the amount of constraints enforced by parameters of a model (e.g., Dynamic Causal Modeling^28^). Contrary to that, recent frameworks offer both a holistic as well as data-driven approach^29,30^. These frameworks provide the possibility to investigate the whole brain on different levels, without the necessity of a predefined set of ROIs or network structure parameters. Of those, the INSIDEOUT approach^29^ relies on the observation of the environment driving hierarchically lower sensory regions stronger than hierarchically higher ones. A system in equilibrium has seamless transitions between different states; thermodynamically, it is reversible in time. Should the system get driven out of equilibrium, the transitions between states become non-reversible and an arrow of time emerges. Measuring the effects of the extrinsic environment on the intrinsic brain dynamics through the non-reversibility of the system, here the brain, can therefore help uncover variations in brain states under different conditions. The framework of normalized directed transfer entropy (NDTE)^30^, contrarily, works on a mesoscopic level: By considering the interconnectivity of all defined brain regions, it draws assumptions about the most essential contributors, or functional hubs, of the underlying networks.

In the current study we investigate the cortical connectivity network underlying the auditory looming bias under the individual factors of cue type and attention, in search for overlapping patterns along spatial and/or identity-related cortical processing streams. Through the high temporal resolution of electroencephalography (EEG) in combination with recently proposed data-driven approaches for connectivity analyses we investigate the brain at different levels of granularity^29,30^: First as a whole, and subsequently in search for the functional hubs that act as essential contributors in the looming network. High spatial resolution is achieved by complementing source localization of high-density EEG with individual brain anatomies and electrode locations^31^.

**Fig. 1:**
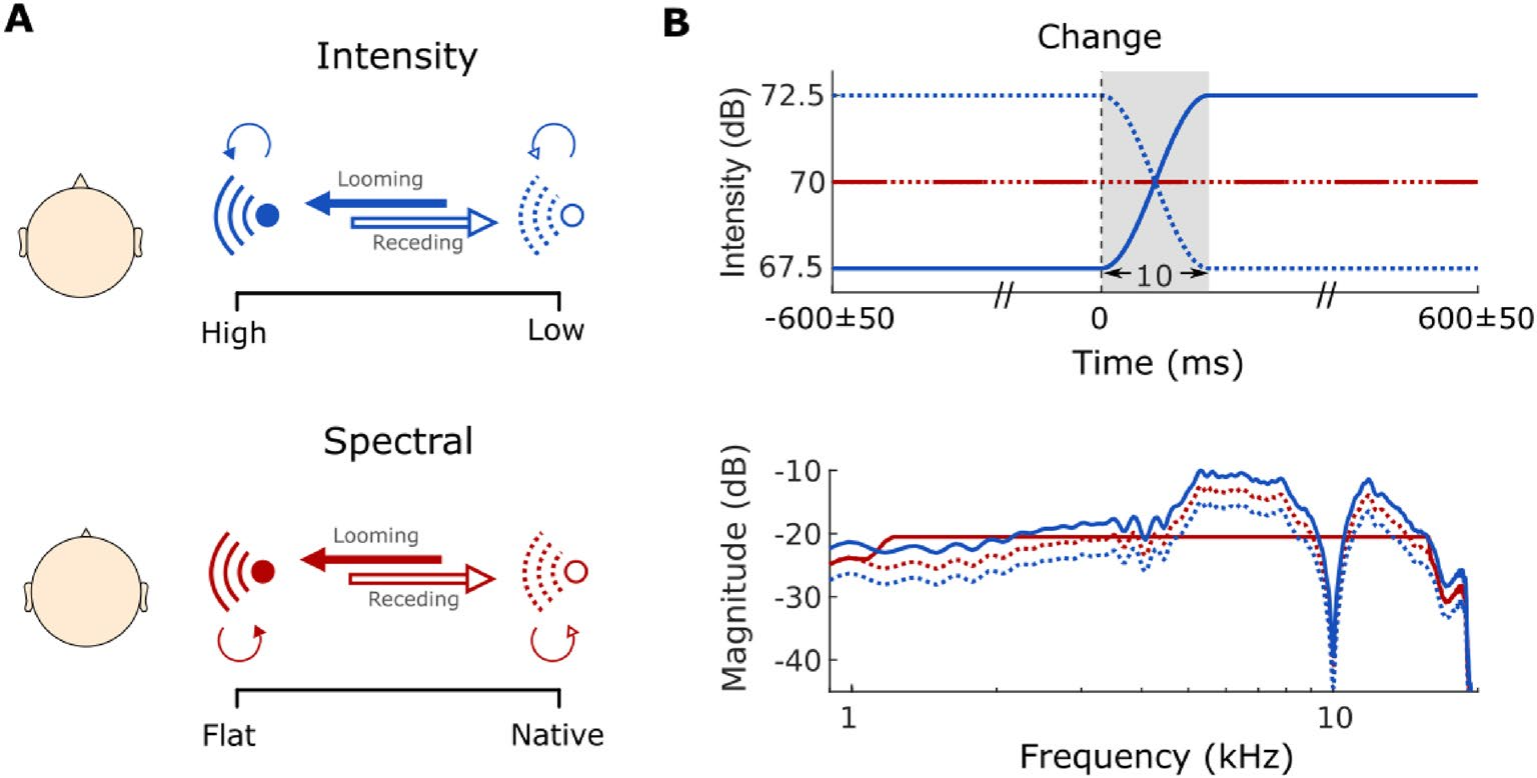
Experimental design. (**A**) Looming and receding percepts created through simulated transition between two sounds of different intensities (top, blue) or spectral shapes (bottom, red). Thick arrows represent $50\%$ transition probability for motion trials (dark = looming; light = receding). (**B**) Sound intensity over time (top panel) and magnitude spectrum (bottom panel) of all implemented stimuli. Figure adapted from^10^.

The present analyses are based on data previously collected by our research group, studying the auditory looming bias at the level of HG under the aforementioned factors of attention and cue type^10^. In that paradigm (Fig. 1), participants listened to broadband harmonic tones that rapidly changed in their simulated distance from the listener and thereby elicited a looming or receding percept (Section “Stimuli and procedure”). Distance cues comprised either overall sound intensity or spectral shape changes. Listeners were first passively exposed to the stimuli while watching a silent subtitled movie and later had to discriminate the sonic motion direction as either static, approaching or receding. Earlier behavioral analyses^10^ revealed that participants detected static sounds with near-perfect accuracy and fast responses, whereas discriminating motion direction was significantly harder and slower. Model-based analyses confirmed a looming bias, with higher drift rates—the rate at which sensory evidence accumulates toward a decision—for approaching than receding sounds across cue types. Accuracies were overall higher for intensity-based cues. Spectral cues, though perceptually more subtle, also elicited a looming bias, and a later study confirmed that they nevertheless induce clear changes in distance perception^32^. In the connectivity analyses presented herein we find higher sensitivity for intensity-based stimuli, while different main hubs, traditionally linked to threat and fear perception, emerge depending on the factors considered.

## Results

For the present connectivity investigations, we extracted the source-localized EEG time series of all cortical regions, as defined by the Desikan-Killiany parcellation^33^. We considered the time interval between 0 and 300 ms relative to the event of distance change. This choice was made based on the finding that this time window has shown significant biases evoked in HG in previous investigations^10^.

### Intensity looms induce stronger non-reversibility in cortical processing

INSIDEOUT reflects how the environment (extrinsic, outside) affects the dynamics and equilibrium of the underlying brain state (intrinsic, inside)^29^, by measuring the non-reversibility of a considered system.

We implemented this framework by accounting for the set of all ROIs of the considered parcellation, hence the cortex as a whole (Section “INSIDEOUT”). Higher non-reversibility is thus understood as a quantification of the amount of change in causal interactions of the brain under each considered condition.

**Fig. 2:**
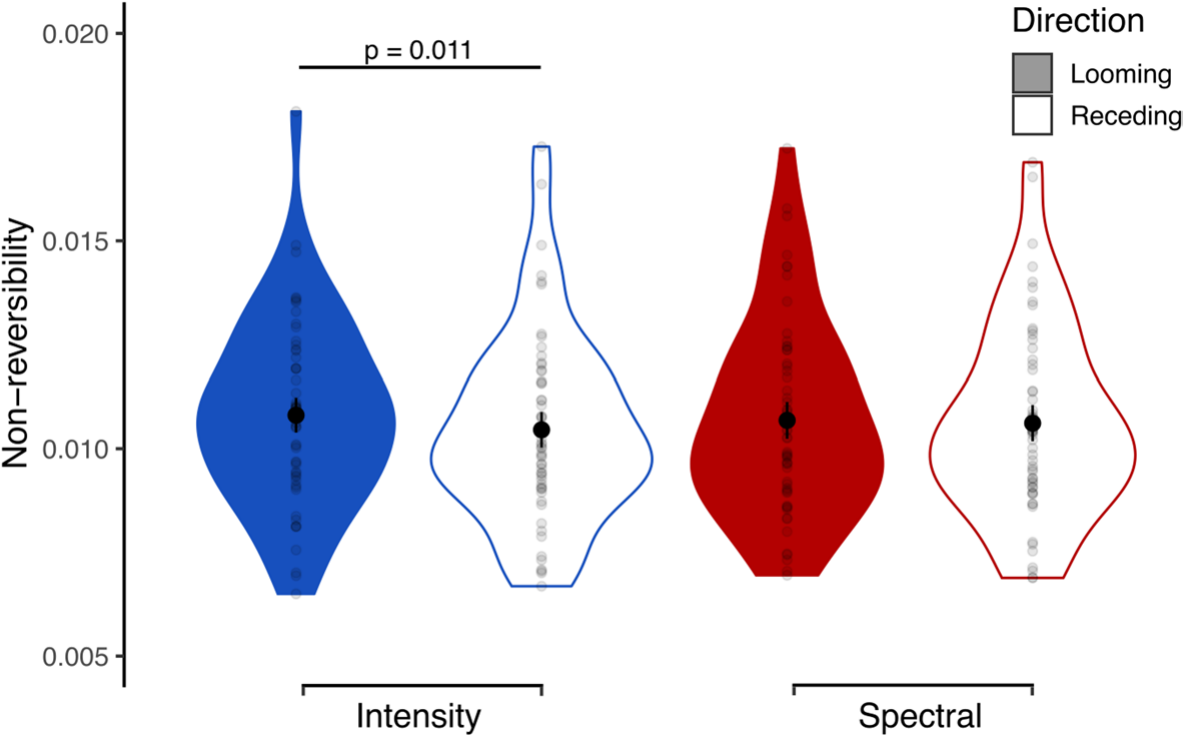
Effects of cue type and movement direction on the temporal non-reversibility of cortical processing, as calculated by the INSIDEOUT framework. Within each violin plot, black points and bars represent the means and their standard errors and gray dots indicate data points for each individual.

Figure 2 shows the distributions of non-reversibility measures obtained for every considered condition. An ANOVA with the factors of attention, cue type and motion direction revealed the latter to be a significant factor ($F_{1,27} = 8.34$, $\eta ^2_p = 0.24$, $p=0.008$), as well as its interaction with the cue type ($F_{1,27} = 5.11$, $\eta ^2_p = 0.16$, $p=0.032$). To further investigate this interaction, we performed a separate ANOVA for each cue type and adjusted the p-values for multiple comparisons using Bonferroni correction. For intensity, looming sounds were found to elicit higher non-reversibility than receding sounds ($F_{1,27} = 9.17$, $\eta ^2_p = 0.25$, $p=0.011$). No significant factors or interactions thereof appeared for the spectral condition. Hence, looming stimuli appeared to disrupt the intrinsic equilibrium more than receding ones, in particular when they were based on intensity changes.

### Connectivity hubs relevant to the auditory looming bias

To better understand the dependencies we then applied the NDTE framework, as it offers a more granular view on the interacting brain regions. Following the procedure suggested in^30^, we considered the connection of each ROI to each remaining ROI in the cortical parcellation. The connectivity between each pair of regions was calculated on the actual data, and its significance was assessed through a distribution of surrogate data stemming from the same ROI-pair (Section “NDTE”). Aggregating the connectivity information across participants allowed for the construction of connectivity hubs, namely regions – or sets thereof – that are, as a whole, more connected compared to any other considered set comprising the same number of ROIs. We performed a connectivity analysis on the bias data by considering the factors of attention and cue type.

The two quantities of essence in this framework are termed inflow ($G_{in}$ in^30^) and outflow ($G_{out}$ in^30^); they respectively represent the connectivity incoming to or outflowing from an ROI. If a set of ROIs is considered as a network, inflow is the sum of all incoming connectivity across all its constituent ROIs. The respective holds for the outflow.

**Fig. 3:**
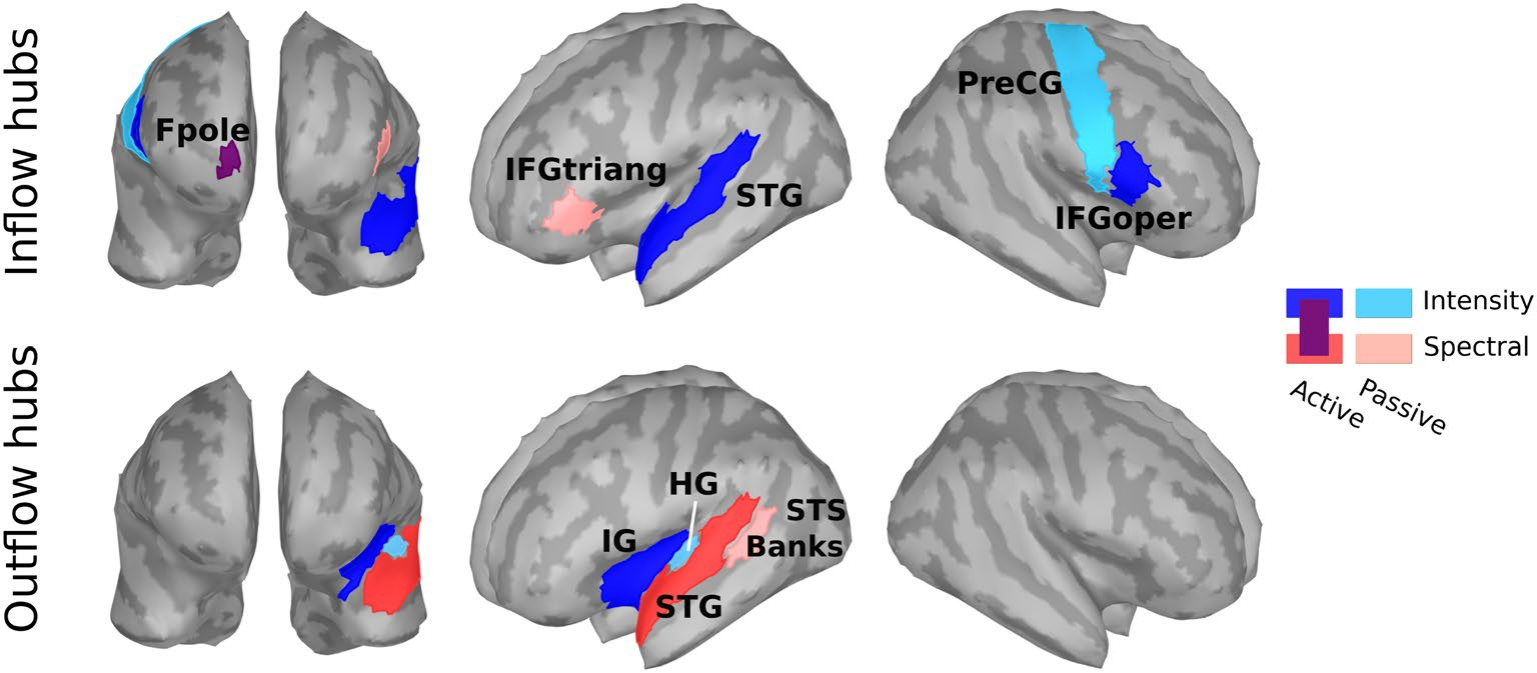
Major inflow and outflow hubs of looming bias identified per considered condition. Fpole (purple) is activated in both active intensity and spectral conditions. Abbreviations: Fpole—frontal pole, IFGtriang—pars triangularis, STG—superior temporal gyrus, PreCG—precentral gyrus, IFGoper—pars opercularis, IG—insular gyrus, BanksSTS—banks of the superior temporal sulcus, HG—Heschl’s gyrus.

We determined the major inflow and outflow hubs per condition by following the concept and search procedure of functional rich clubs (FRICs in^30^). Following the procedure for their definition based on the inflow, we respectively defined the major hubs based on the connectivity outflow. As shown in Fig. 3, the ROIs receiving the most inflow spanned over temporal regions (STG), frontal regions (pars opercularis – IFGoper, frontal pole – Fpole) and both hemispheres. Across both active conditions, only the frontal pole emerged as a crucial inflow hub for the looming bias. In the passive conditions, the relevant inflow hubs comprised the right precentral gyrus (PreCG) for intensity and the left pars triangularis (IFGtriang) for spectral stimuli. Regarding the outflow hubs, one region emerged per condition and all regions were located in the left hemisphere. Apart from the active intensity condition, where the insular gyrus (IG) was identified as the main hub, temporal regions were identified for the remaining cases: superior temporal gyrus (STG) for active spectral, HG (defined as transverse temporal gyrus in the Desikan-Killiany atlas) for passive intensity, and the banks of the superior temporal sulcus (BanksSTS) for passive spectral.

**Fig. 4:**
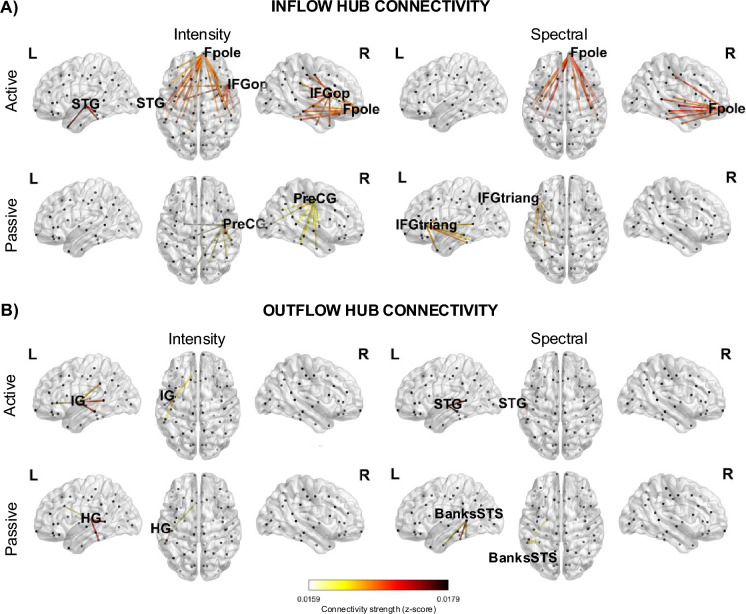
Detailed connectivity patterns of major inflow and outflow hubs identified for each condition. Only the top 30% of connectivity strengths per condition are displayed to enhance visual clarity. Line color denotes connectivity strength, and black dots mark the ROIs. Hub ROIs are labeled according to the anatomical atlas labels introduced in the caption of Fig. 3.

We further extracted the pattern of hub connections that emerged, separately for inflow and outflow connectivity in each considered condition (Fig. 4). On a large scale, the inflow hubs, localized to the frontal cortices (Fpole, IFG, PreCG), dominantly received information bilaterally from more distant regions of the sensory temporal regions such as STG, HG and inferior temporal cortices. In contrast, the outflow hubs, localized to the temporal regions (IG, STG, HG, STS), tended to send information to more local areas within the temporal cortex.

## Discussion

Looming sounds exhibit remarkable salience, consistently eliciting a perceptual bias compared to equivalent receding sounds. This bias is commonly hypothesized to signify a mechanism for threat detection and hazard protection. In this study we employed two data-driven, state-of-the-art functional connectivity approaches to examine cortical responses to simulations of rapid changes in auditory distance. Distance changes were cued utilizing either intensity or spectral shape, and we examined the responses during both passive and active listening. On the level of macroscopic brain states, the overall functional connectivity, represented by temporal non-reversibility values, was more susceptible to the intensity-evoked looming percept. Taking a more granular approach, we identified functional connectivity hubs that varied considerably across conditions. Table 1 lists each major in- and/or outflow hub we identified, along with its likely stream/network memberships as well as primary and threat-related functions. In the following we discuss each of the observed activations and condition-specific shifts in light of the known functions of the identified regions and networks.

**Table 1:** ROI hubs, putative stream/network memberships, functions and interpretive notes. Abbreviations: HG—Heschl’s gyrus, STG—superiortemporal gyrus, BanksSTS—banks of the superiortemporal sulcus, IFGoper—inferior frontal gyrus pars opercularis, IFGtriang—inferior grontal gyrus pars trriangularis, Fpole—frontalpole, BA—Brodmann area, PreCG—precentral gyrus, IG—insular gyrus, DMN—default mode network.

**Hub ROI**	**Condition**	**Auditory stream**	**Network**	**Primary functions**	**Interpretation**
left **HG**	Outflow passive intensity	Ventral “what” (core auditory input)	Auditory/language system	Fine spectrotemporal analysis and envelope coding^39,40^	Provides sensory evidence to frontal nodes; looming advantage via rapid intensity encoding
left **STG**	Inflow active intensity, outflow active spectral	Ventral “what” with motion links	Auditory/language; multimodal association	Complex spectral patterning and auditory object/motion processing^39,50^	Outflow hub for object/salience formation; stronger for looming intensity cues
left **BanksSTS**	Outflow passive spectral	Ventral “what” (polymodal)	Audiovisual association	Integration of spectral regularities and complex sound prediction^39,51^	Emphasized for spectral looming (identity-rich), supporting predictive mapping to IFG
left **IFGtriang**	Inflow passive spectral	Ventral “what” / language interface	Frontoparietal control, language system	Categorical decision and semantic/predictive coding^17,52,53^; threat/fear responses^41–44^	Evaluates identity-like spectral cues signaling threat without response demands
right **IFGoper**	Inflow active intensity	Ventral “what” / sensorimotor bridge	Frontoparietal control / dorsal premotor	Sensorimotor rule mapping, response selection and timing^17,53,54^; threat/fear responses^41–44^	Integration of intensity-looming urgency with action rules, replacing passive PreCG; active threat response to looming urgency
right **Fpole (BA10)**	Inflow all active	None (supramodal)	Frontoparietal control	Evidence integration, exploration–exploitation, task set^53,55^	Shift from IFGtriang (passive) to Fpole (active) reflects added control/decision demands
right **PreCG**	Inflow passive intensity	Dorsal audio–motor (not classic “where”)	Sensorimotor / orienting	Motor readiness; preparatory orienting to salient onsets^37,38^	Intensity-looming evokes automatic orienting in passive listening; replaced by IFGoper when active
left **IG**	Outflow active intensity	None (salience gateway)	Salience network	Salience detection and network switching (DMN $\longleftrightarrow$ control)^48,49^; fear and anxiety^43,47^	Intensity-based looming strongly engages salience network; IG disseminates “urgency” to control systems triggering fear-related response

### Condition-specific activity within cortical networks

Among the frontal regions, the anterior part of the right frontal cortex (Fpole, BA10) emerged as the major inflow hub in both active conditions. Its activity can indicate the recruitment of the frontoparietal control system for evidence integration and action selection under task demands, consistent with the region’s role in higher-order control and decision-making^34–36^. The presence of right PreCG as an inflow hub for the passive intensity condition is compatible with fast audio–motor orienting/readiness to act in response to approaching cues^37,38^. Lastly, the shift to left IFGtriang during passive exposure to spectral cue changes aligns with the ventral auditory “what” stream (source identity) and language-related predictive coding mechanisms operating on complex spectral structure^39,40^. The IFG sits at the intersection of the ventral auditory stream and the frontoparietal control network, supporting rapid categorization and remapping of salient sound events to action rules. IFG activity is often associated with threat and fear responses. For instance, studies of visually presented threat-related words have reported activation of the left IFG^41,42^. It has been further activated in the context of fear conditioning^43^ and downregulation of psychophysiological reactions to threat^44^. The importance of source identity on the elicitation of auditory looming bias has previously also been demonstrated by comparisons between different types of source stimuli, where tones elicited stronger biases than noise^4,5,9,45,46^. The here identified connectivity hubs seem to reflect the crucial role of source identity in the manifestation of the auditory looming bias.

The insula, traditionally associated with fear and anxiety conditioning^41,43,47^, emerged as the principal outflow hub in the active intensity condition. As an ROI it is moreover crucial in the engagement of the salience network^43,48^, which is well known to detect behaviorally relevant events and orchestrate switching between default and control networks^49^. In our paradigm, intensity-based looming provides a strong, low-level urgency signal, which dovetails with our whole-cortex INSIDEOUT result showing greater non-reversibility for looming versus receding only for intensity cues. Intensity stimuli have behaviorally and neurally emerged as more salient than their spectral counterparts in both adult and newborn listeners^10^. In line with this, we construe our result as a macroscopic signature of enhanced environmental drive and arousal. This macro-scale state-change measure supports the interpretation that intensity-driven looming is more “salience-weighted” than spectrally driven looming.

The information received by the major inflow hubs mainly appeared to originate from primary and secondary auditory cortical regions in the temporal lobe. Temporal outflow hubs (HG/STG/STS) across conditions are situated within the ventral auditory stream; they conduct fine spectrotemporal analysis of sensory evidence^56^ and provide its feedforward dissemination toward frontal control areas^39,40^. The emerged predominance of left-hemisphere temporal outflow may signal the left auditory cortex’s relative advantage for rapid temporal-envelope coding^57^ and its tight coupling with left IFG during categorical decisions on complex sounds. Prior studies have argued about the directionality of the connectivity in the looming bias, with results divided between bottom-up processing^13^ or a top-down intervention^23^. The here-identified hubs support the bottom-up notion. Consistent with the notion of threat-related bias activation, previous research has implicated HG in the processing of threatening sounds^41^. Superior temporal ROIs, similar to those emerging in our active (attentive) conditions, have moreover been associated with attention to threatening voices^58^.

Importantly, we did not find robust hubs classically associated with the dorsal auditory “where” stream (e.g., IPL and intraparietal sulcus^59^) in the looming-minus-receding contrasts. We speculate that the subtraction likely canceled spatial-trajectory computations shared by both motion directions and that our $0-300$ ms analysis window emphasizes early object/salience processing over later spatial updating. Although a potential limitation, this can be leveraged as a testable prediction for future work using longer windows or contrasts that specifically isolate spatial tracking.

As we conducted our study based on EEG data, subcortical activations are either inaccessible or unreliable; yet, for completeness, we would like to touch upon a possible subcortical source of our results: Frontotemporal activations have generally been linked to the basolateral amygdala (BLA), an essential hub of the limbic system, in the context of automatic fear detection^60^. The amygdala itself has, in turn, been further implicated in looming perception as part of a warning mechanism^14^. The direct verification of the BLA-frontotemporal link in the context of the bias can not be made through our findings. Yet, the emerging frontal and temporal connectivity hubs may be a manifestation of the BLA-frontotemporal exchange. Invasive studies on animals have specifically implicated the medial prefrontal cortex and BLA in the discrimination between harmful and safe stimuli, and highlighted that the corticocortical dialogue between sensory and prefrontal areas is essential for fear-discrimination processes^61^.

In summary, active listening imposes explicit decision requirements, recruiting Fpole and the broader frontoparietal control system to integrate sensory evidence and map it onto responses. Passive listening to spectral cues, which are acoustically subtler and identity-rich, leans more on ventral-stream interfaces in IFGtriang. For intensity cues, the passive condition’s PreCG involvement likely reflects automatic preparatory orienting to approaching sound energy, whereas in the active task IFGoper is better positioned to couple ventral auditory evidence with response selection. Looming carries higher behavioral urgency and more reliably engages rapid, partly automatic orienting. This may yield stronger coupling with the salience network and faster ventral-stream–to–control-network handoffs. This is particularly important for intensity-based looming, as it likely provides a robust physical urgency cue. Spectral looming, while still biasing perception toward approach, relies more on learned spectral-object regularities and thus engages ventral temporal regions and IFG in a way that is less arousal-driven and more identity-evaluative. This framework may clarify why we see insular outflow predominantly with intensity and IFG/frontopolar inflow across active conditions; it additionally accommodates the observed left-hemisphere bias in outflow hubs (temporal ventral stream linked to IFG) and underscores the threat relationship of looming responses.

### Methodology and limitations

Despite the above evidence for eliciting threat-related neural activity, we acknowledge that our study did not include direct behavioral or subjective measures of threat perception. Instead, we rely on prior evidence showing that looming sounds elicit higher subjective ratings of threat and increased skin conductance responses compared to receding sounds^62^, and we follow the predominant view that auditory looming bias constitutes a threat-relevant cognitive bias^63,64^. Previous work has linked looming-related biases to threat processing more broadly, including preferential encoding in memory and attention^65^. Importantly, this bias is amplified in states of physical^66^ or mental vulnerability^67,68^, supporting its functional relation to perceived threat. In line with this interpretation, the behavioral data from the original study^10^, on which the present work builds, showed that sensory evidence for looming sounds is accumulated more rapidly (i.e., showing higher drift rates) than equivalent evidence for receding sounds, consistent with the notion of threat-related urgency. Due to that, the distinction between threat-related and more general arousal-related processes cannot be drawn conclusively at this stage^62,69^. Future studies are needed to explicitly disentangle these emotional dimensions; for instance, by contrasting fear vs. reward cues and assessing arousal via physiological markers such as skin conductance^70^.

In the current study, we utilized direct (NDTE) and indirect (INSIDEOUT) connectivity metrics in order to obtain an image of the bias-related processes on the cortical surface. Depending on the method at hand, investigations can be done at different levels of granularity. INSIDEOUT captures the breaking of causal connections through non-reversibility and the arrow of time in order to measure brain connectivity. Compared to other approaches, it has the big advantage that no underlying constraints (e.g., preselection of ROIs or networks) or models (e.g., directionality or node assumptions) are necessary for its implementation. It can additionally give a coarse representation of the different brain states based on the whole cortex in a significantly less computationally complex and time-consuming manner than conventional approaches would demand. In terms of non-reversibility, the looming bias was found mainly for the intensity stimuli. Broadly considered in looming studies, intensity stimuli have generally appeared more salient than spectral ones; the latter seem to be more complex in their understanding and cognitively processed in a much more subtle manner^71–73^. As INSIDEOUT is reflective of subjective conscious awareness^29^, our result corroborates the difference in perception depending on cue type. The greater intervention of intensity stimuli, in terms of disruption in causal interactions, highlights their salience as already emerged through prior behavioral as well as neural studies^6,7,9,10,12,14,15,23,64,74^.

Contrary to the coarse granularity offered by INSIDEOUT, the fine-grained method of NDTE yielded insights into which regions are the main hubs in manifesting the looming bias, and does so in a data-driven way. By considering all ROIs of a given parcellation, the cross-connectivity is calculated. We obtained a limited numerical range of connectivity strengths (e.g., differences in the order of 0.05); those values represent normalized (z-scored) strength measures derived from group-averaged, threshold-controlled networks. Under this normalization, small absolute differences are typical^29,30^ and are nevertheless reliable at the group level as confirmed through permutation testing. By, then, ranking regions based on their outflow (sources) or inflow (receivers) and iteratively comparing networks, conclusions about ROIs, or networks thereof, with the most essential contribution per considered condition emerge. It should be noted that the timescale of all effects is defined by the calculated minimum of the autocorrelation function. As shown in previous research, this is a solid approach to our investigations^29,30^. In a more ideal way, though, and although computationally significantly more costly, this parameter could be set individually for each considered time series.

In our investigation we adhered to the rather coarse parcellation of the Desikan-Killiany atlas^33^. Our selection relies on both aiming to compare outcomes to prior literature^13,23^ as well as reduce complexity, especially in the case of NDTE calculations. Finer parcellations, such as the one from Destrieux^75^, could offer different insights depending on the question at hand; yet they come with higher amount of regions and therefore computational complexity. Importantly, finer parcellations are more prone to wrongful activity attribution: In our study we used EEG, which notoriously suffers from spatial imprecision due to conductivity and diffusion effects from the layers between the cortex and the sensors. Although we used individualised anatomical information to aid the performed cortical source localization as much as possible^31^, spatial imprecisions of activity allocation are inevitable. An example thereof is the depth-weighting done by algorithms for sources that are intricately placed on the cortex. Additionally, should activity arise from subcortical surfaces at greater distances from the sensors, EEG may wrongfully attribute the recorded activity to the cortical brain sources. We therefore decided against a finer parcellation for our study, considering that results can possibly be misleading. Our results are in good agreement with relevant literature, yet different imaging methods, selected parcellations or implemented algorithms may lead to slightly altered outcomes.

## Methods

### Participants

Thirty-five healthy young adults were invited for study participation. Exclusion criteria during recruitment comprised self-reported indications of psychological and neurological disorders or acute or chronic heavy respiratory diseases that may prevent the participant from sitting still during the EEG recording.

All invited participants signed informed consent prior to testing, were neither deceived nor harmed in any way and were informed that they could abort the experiment at any time without any justification or consequences. The study was conducted in accordance with the standards of the Declaration of Helsinki (2000). No additional ethics committee approval was required given the non-medical non-invasive nature of our study, as per the Austrian Universities Act of 2002. In total, experiments lasted around five hours per participant and participants received monetary compensation in return for their time.

Prior to conducting the experiment, participants’ hearing thresholds between 1 and 12.5 kHz were measured via pure tone audiometry (Sennheiser HDA200; AGRA Expsuite application^76^), with a deviation of more than 20 dB from the age mean^77^ leading to participant exclusion. Six participants were excluded (29 remaining participants, 15 females: $25.0 \pm 2.60$ years old (mean ± standard deviation); 14 males: $25.1 \pm 2.77$ years old). An error rate in recognition of static sounds (catch-trials) exceeding $20\%$ resulted in one additional exclusion (female, $45.2\%$ errors). In total 28 participants were included in this study.

### Stimuli and procedure

The experiment consisted of two phases: an initial passive listening phase and a subsequent active phase. During the passive listening phase, participants watched a silent, subtitled movie to divert their attention, while they were passively exposed to the auditory stimuli without any task demands. In the active listening phase, participants engaged in a three-alternative motion discrimination task (3-AFC), adapted from a previous study^12^. In this task they categorized each stimulus as looming, receding, or static using three designated keyboard keys.

Trials were randomized throughout the experiment and balanced over blocks, with $50\%$ moving and $50\%$ static trials. Static sounds served two key functions: they prevented participants from anticipating the stimulus type based on its onset, as static sounds shared identical onsets with moving stimuli but lacked the transition; and they acted as catch trials to discourage random responses throughout the experiment. Moving trials comprised $50\%$ looming and $50\%$ receding sounds, created by either modifying the sound’s intensity (Fig. 1B, top, blue curves) or spectral shape (Fig. 1B, bottom, red curves) and crossfading between the final simulated sound source positions (from far to near for the looming, and near to far for the receding condition). The onset of the transition phase—the ’change event’—was temporally jittered by 50 ms to reduce predictability. The transition itself was kept brief (10 ms) to ensure high temporal resolution in analyzing neural responses to the change event.

The intensity manipulation resulted in the sound’s intensity varying over time, while its spectral content remained constant, thus producing a simple change in overall level. We presented sounds crossfading between $+2.5$ dB (near position) and $-2.5$ dB (far position) to induce looming and receding sensations^11^. For changes in spectral shape, we followed the procedure introduced in^12^ and manipulated the head-related transfer functions (HRTFs), individually recorded at a distance of 1.2m; those filters describe the acoustic effects of the pinnae, head, and torso. In contrast to intensity stimuli, spectral stimuli maintained a constant broadband level but changed in spectral content between a flat spectrum and listener-specific HRTFs. This distinction was crucial for isolating the more salient intensity cues from more complex, less accessible spectral ones. The different cue types were applied blockwise. Apart from movement and spatial cue type, we block-wise manipulated whether the sound source was presented from the left or the right side of the listener.

All auditory stimuli were complex harmonic tones^78^ ($F_0 = 100$ Hz, bandwidth $1-16$ kHz), filtered with the listener-specific HRTFs to create the percept of a source on either the left or right side when presented over earphones (ER-2 insert earphones, Etymotic Research Inc., Grove Village, Illinois). Stimulus duration was 1.2 s with 10 ms onset and offset ramps of raised-cosine shape. Inter-stimulus intervals lasted 500 ms in the passive listening condition.

Stimuli and procedures were programmed in MATLAB (R2018b, Mathworks, Natick, Massachusetts) using the Auditory Modeling Toolbox^79^ and Psychtoolbox^80^.

### Recordings and processing

EEG recordings were done with a 128-channel system (actiCAP with actiCHamp; Brain Products GmbH, Gilching, Germany) at a sampling rate of 1 kHz. Noisy channels were being noted during the recordings. All saved EEG data were visually inspected to detect potential additional noisy channels, which were then spherically interpolated. Inspected data were bandpass-filtered between $0.5-100$ Hz (Kaiser window, $\beta = 7.2$, $n= 462$) and epoched ($[-200, 1500]$ ms) relative to stimulus onset. We applied hard thresholds at $-200$ and $800 \ \mu V$ to detect and inspect extremely noisy trials. An additional check for the identification of additional bad channels was implemented, via an automatic channel rejection step; detected channels would then be visually inspected and interpolated. No additional noisy channels were detected for any of the participants at this step. Independent component analysis (ICA) was followed by a manual artifact inspection and rejection of oculomotor artifacts (removal of up to 3 components per participant). The cleaned data were thereafter re-referenced to their average. Within each participant, trials were equalized to match the condition with the minimum amount within the participant after trial rejection. This was achieved by pseudo-selection, aiming to maintain an equal distribution across the recordings and resulted in an average of 569 clean trials (SD $= 27.7$) per participant. All preprocessing steps were undertaken in the EEGLAB free software^81^ in MATLAB (R2018b).

Twenty-five (25) of 28 participants had their individual anatomical structures and electrode positions recorded. Anatomical magnetic resonance images (MRIs) were segmented via Freesurfer^82^ and used to create a study protocol on Brainstorm^83^. For the remaining 3 participants, the default anatomical models of Brainstorm were used (ICBM152 brain template); individual MRIs could not be recorded due to incompatibilities with the scanner (suspicion of metallic parts in the body). Anatomical models were created via OpenMEEG^84^ with following parameters: boundary element model (BEM) surfaces had 1922 vertices per layer for scalp, outer skull and inner skull, and a skull thickness of 4 mm. The relative conductivity was set to 0.0125 for the outer skull and to 1 for the remaining layers. Manual co-registration between head model and individual electrode locations was done for each participant individually. Recorded activity was inferred to the cortical surface via dynamic statistical parametric mapping (dSPM)^85^. The noise covariance was calculated from a 200 ms pre-stimulus interval. Dipole orientations were considered constrained to the surface and source signals were reconstructed at 15000 vertices describing the pial surface. Following previous literature^13,23^, cortical mapping was done according to the Desikan-Killiany parcellation^33^. We extracted all ROI time series from the 68 areas of the atlas, as defined in Brainstorm. Based on the evoked time courses at the level of the transverse temporal gyrus, taken from^10^, a time window of 300 ms post-change was defined as the time window of interest.

### Connectivity calculations

Our NDTE connectivity analyses, being based on Granger causality, assume stationary signals as input. In order to fulfill this stationarity requirement, we tested our time courses for this property. Following the recommendations of Brainstorm^83^, each time-series was subjected to both the Kwiatkowski-Phillips-Schmidt-Shin test (KPSS) for trend-stationarity and the unit root Augmented Dickey Fuller test (ADF), as implemented in MATLAB (R2018b, kpsstest, adftest). As broadband EEG signals are highly non-stationary, stationarity of all signals was restored through double differencing of the individual time-series^24^.

#### INSIDEOUT

The INSIDEOUT framework^29^ is based on the time-shifted correlation matrices between each considered time series and its time-reversed version, thereby echoing the asymmetry in temporal processing. The arrow of time captures the interaction with the environment: A system that remains unperturbed by external factors maintains its intrinsic equilibrium and is therefore characterised by high reversibility. Higher dissimilarity of the forward and reverse time series corresponds to higher non-reversibility, and thereby higher impact of the external environment on the intrinsic dynamics.

Reversed time series were obtained by inverting the original ones in time for each condition, participant, ROI and trial. Correlations between time series were calculated through the MATLAB (R2018) function corr, for both the forward as well as the reverse time-shifted correlations. If $FS_{forward}(T)$ and $FS_{reversal}(T)$, expressed as mutual information based on the time-shifted correlations, are the matrices representing the causal dependencies of the system, here across ROIs, the non-reversibility (non-equilibrium) per condition is calculated as

1 $$\begin{aligned} NR = ||FS_{forward}(T) - FS_{reversal}(T)||_2 \end{aligned}$$

and is hence equal to the mean of the absolute squared difference between the forward and reversed matrices (cf.^29^ for detailed calculations). Time-shift T is defined as the decay to the first minimum of the autocorrelation function across conditions and participants^29,30^.

Statistical differences among the conditions were assessed based on ANOVA with the factors of attention, cue type and motion direction.

#### NDTE

The data used in the assessment of NDTE were based on the extracted looming bias on a single trial basis for each ROI. All subsequent calculations were done following the pipeline described by Deco et al.^30^. In it, the statistical causal interaction between any two ROIs is assessed based on the measure of mutual information. Considering *X* and *Y* to denote the activity of the source and target ROIs respectively, the mutual information is calculated as

2 $$\begin{aligned} I(Y_{i+1};X^{i}|Y^{i}) = H(Y_{i+1}|Y^{i}) - H(Y_{i+1}|X^{i},Y^{i}) \end{aligned}$$

where $I(Y_{i+1};X^{i}|Y^{i})$ corresponds to the degree of statistical dependence between the source’s past $X^{i}=[X_i,X_{i-1},...,X_{i-(T-1)}]$ and the target’s immediate future $Y_{i+1}$^30^. $H(Y_{i+1}|Y^{i})$ and $H(Y_{i+1}|X^{i},Y^{i})$ express the respective conditional entropies, that are, in the implemented framework, estimated based on covariance matrices^86^. The time interval defined by *T* stems from the autocorrelation of the time series. Following Deco et al.^30^, the corresponding order (“maximum lag”) was calculated based on the decay to the first minimum of the autocorrelation function across conditions and participants; in our case $T=6$. In order to be able to compare and combine NDTE values across ROI pairs, calculated connectivity values were normalised as

3 $$\begin{aligned} F_{XY} = I(Y_{i+1};X^{i}|Y^{i})/I(Y_{i+1};X^{i},Y^{i}) \end{aligned}$$

where the normalization factor $I(Y_{i+1};X^{i},Y^{i})$ represents the total mutual information the past of both source *X* (here $X^{i}$) and target *Y* (here $Y^{i}$) hold about the future of target *Y* (here $I(Y_{i+1}$). This is the quantity considered throughout our calculations and yields, for each trial, an NDTE matrix with the bidirectional flow among all 68 ROIs of the parcellation.

As the whole cortical surface and all bidirectional connections therein are considered, the high amount of comparisons is susceptible to spurious correlation outcomes. For that reason, circular-shift surrogate data were generated for each considered ROI pair. P-values for each connection were assessed based on the distribution of connectivity data resulting from 100 independent circular time-shifted surrogate iterations. Statistical significance of connections between ROIs was calculated through p-value aggregation done by Stouffer’s method^87^ in two steps; initially at a participant level with a within-condition aggregation across trials of a participant, and subsequently at a group level, with aggregation across all participants of a condition. For each considered condition, the multiple comparison correction was performed by the false discovery rate method (FDR)^88^. The corrected values were then used as a binary “significance mask”, to select the significant connections per condition. The resulting data comprised one NDTE matrix of dimensions ROI x ROI per condition, containing the averaged connectivity values for the ROI pairs that survived the significance evaluations. Inflow ROIs are positioned along the first, while outflow ROIs along the second dimension of the NDTE matrix, termed $C_{All}$.

For each ROI *i* of $C_{All}$, the total inflow from all remaining ROIs *j* of the cortical parcellation is defined as the sum of connectivity across all columns of the matrix: $G_{in}(i) = \Sigma _j C_{{All}_{i,j}}$. The respective holds for the total outflow per ROI *j*: $G_{out}(j) = \Sigma _i C_{{All}_{i,j}}$.

The major hubs are identified through an iterative process. After sorting the regions based on their inflow (for inflow hubs) or outflow (for outflow hubs), an algorithm searches for the largest subset of ROIs *k* that have a value $G_{hub}$ significantly larger than any other set comprising the same amount of regions. The significance value of each $G_{hub}(k)$ is assessed via 1000 Monte Carlo simulations, where for each permutation, one member of the current subset *k* is substituted with any of the remaining ones from the parcellation, and the $G_{hub}(k)$ is calculated anew. The in- and outflow values are calculated as

4 $$\begin{aligned} G_{hub}(k) = \Sigma _k C_{{All}_k} + a*\Sigma _k G_{in}(k) - b*\Sigma _k G_{out}(k) \end{aligned}$$

where $\Sigma _k C_{{All}_k}$ is the total flow within the considered subset, $\Sigma _k G_{in}(k)$ represents the total inflow to the considered subset from all ROIs of the parcellation and $\Sigma _k G_{out}(k)$ the total outflow of the subset to the rest of the ROIs. For the inflow hubs the mutlipliers are $[a=1; b=1]$ and for the outflow hubs $[a=-1; b=-1]$.

By progressively adding one ROI (“node” in Deco et al.^30^) to the considered subset ($k = [i_1,...,i_l]$, where *l* is the whole set of ROIs), the major hubs emerge as the set for which the in- or outflow is still within significance limits (i.e., smaller than 0.05).

## Data Availability

Data are available under https://osf.io/4gdy2/.
